# Objective critical appraisal of mammography images in clinical audit: can we achieve this?

**DOI:** 10.1186/bcr2955

**Published:** 2011-11-04

**Authors:** D O'Leary, A Teape, J Hammond, L Rainford

**Affiliations:** 1 UCD, School of Medicine and Medical Science, Dublin, Ireland; 2 BreastCheck Irish National Breast Screening, Dublin, Ireland

## Introduction

Objective critique of mammographic image quality (IQ) is vital to assess efficacy of services provided by mammography units. The European Guidelines for Screening Mammography require an inadequate image rate of 3%. The subjectivity within current IQ criteria allows symptomatic mammograms with surgically altered breasts to appear to achieve these requirements; however, as evidenced in the analysis of symptomatic breast images within a national optimisation study, current methods of classification for symptomatic mammograms are largely subjective, resulting in poor inter/intra-departmental agreement on IQ.

## Methods

The European Quality Criteria for mammographic IQ and the Breast Screening quality criteria classification of images as inadequate/moderate/good/perfect were modified to remove all subjective criteria. These objective classifications of IQ were tested for inter/intrarater reliability by a panel of experts and compared with original IQ criteria. Further objective measures such as breast volume, density and pectoral-nipple measurements were carried out.

## Results

When tested with 278 surgically modified breast images from the larger research sample, inter-rater reliability (*K *> 0.701; *P *< 0.001) and agreement (Pearson's correlation *r *> 0.884; *P *< 0.01) by the evaluation panel were higher than when the original quality criteria methods were used. The intra-rater reliability was equally high (*K *> 0.7; *P *< 0.001) with agreement via Pearson's correlation at *r *> 0.844; *P *< 0.01.

## Conclusion

A method of scoring images combining the most objective components of major European, national and international image scoring systems is suggested. The removal of subjectivity from the scoring systems will remove all doubt regarding the achievement of high image-quality goals for all mammography departments.

